# Varying *Flavobacterium psychrophilum* shedding dynamics in three bacterial coldwater disease-susceptible salmonid (Family *Salmonidae*) species

**DOI:** 10.1128/spectrum.03601-23

**Published:** 2023-12-19

**Authors:** Christopher Knupp, Esteban Soto, Thomas P. Loch

**Affiliations:** 1 Michigan State University-Aquatic Animal Health Laboratory, East Lansing, Michigan, USA; 2 Department of Fisheries and Wildlife, College of Agriculture and Natural Resources, Michigan State University, East Lansing, Michigan, USA; 3 Department of Medicine and Epidemiology, School of Veterinary Medicine, University of California, Davis, California, USA; 4 Department of Pathobiology and Diagnostic Investigation, College of Veterinary Medicine, Michigan State University, East Lansing, Michigan, USA; University of Guelph College of Biological Science, Guelph, Ontario, Canada

**Keywords:** bacterial coldwater disease, rainbow trout fry syndrome, *Flavobacterium*, shedding, transmission, disease ecology

## Abstract

*Flavobacterium psychrophilum* causes bacterial coldwater disease (BCWD) and is responsible for substantial losses in farm and hatchery-reared salmonids (Family *Salmonidae*). Although *F. psychrophilum* infects multiple economically important salmonids and is transmitted horizontally, the extent of knowledge regarding *F. psychrophilum* shedding rates and duration is limited to rainbow trout (*Oncorhynchus mykiss*). Concurrently, hundreds of *F. psychrophilum* sequence types (STs) have been described using multilocus sequence typing (MLST), and evidence suggests that some variants have distinct phenotypes, including differences in host associations. Whether shedding dynamics differ among *F. psychrophilum* variants and/or salmonids remains unknown. Thus, three *F. psychrophilum* isolates (e.g., US19, US62, and US87) in three MLST STs (e.g., ST13, ST277, and ST275) with apparent host associations for coho salmon (*O. kisutch*), Atlantic salmon (*Salmo salar*), or rainbow trout were intramuscularly injected into each respective fish species. Shedding rates of live and dead fish were determined by quantifying *F. psychrophilum* loads in water via quantitative PCR. Both live and dead Atlantic and coho salmon shed *F. psychrophilum*, as did live and dead rainbow trout. Regardless of salmonid species, dead fish shed *F. psychrophilum* at higher rates (e.g., up to ~10^8^–10^10^ cells/fish/hour) compared to live fish (up to ~10^7^–10^9^ cells/fish/hour) and for a longer duration (5–35 days vs 98 days); however, shedding dynamics varied by *F. psychrophilum* variant and/or host species, a matter that may complicate BCWD management. Findings herein expand knowledge on *F. psychrophilum* shedding dynamics across multiple salmonid species and can be used to inform future BCWD management strategies.

**IMPORTANCE:**

*Flavobacterium psychrophilum* causes bacterial coldwater disease (BCWD) and rainbow trout fry syndrome, both of which cause substantial losses in farmed and hatchery-reared salmon and trout populations worldwide. This study provides insight into *F. psychrophilum* shedding dynamics in rainbow trout (*Oncorhynchus mykiss*) and, for the first time, coho salmon (*O. kisutch*) and Atlantic salmon (*Salmo salar*). Findings revealed that live and dead fish of all fish species shed the bacterium. However, dead fish shed *F. psychrophilum* at higher rates than living fish, emphasizing the importance of removing dead fish in farms and hatcheries. Furthermore, shedding dynamics may differ according to *F. psychrophilum* genetic variant and/or fish species, a matter that may complicate BCWD management. Overall, study results provide deeper insight into *F. psychrophilum* shedding dynamics and will guide future BCWD management strategies.

## INTRODUCTION

*Flavobacterium psychrophilum*, causative agent of bacterial coldwater disease (BCWD) and rainbow trout fry syndrome (RTFS), causes substantial losses in farmed and hatchery-reared salmonid species (Family *Salmonidae*) (1). Many salmonid species are BCWD susceptible (2), and although rainbow trout (*Oncorhynchus mykiss*) and coho salmon (*O. kisutch*) are generally considered most at risk (3), BCWD in Atlantic salmon (*Salmo salar*) is also common (4–6). Trout and salmon naturally infected by *F. psychrophilum* in their early life stages sustain substantial losses, with mortality rates ranging from 20% to 90% (7, 8). Although vertical transmission of *F. psychrophilum* plays an important role in the perpetuation of BCWD (9–12), horizontal transmission is also problematic (2), facilitating the spread of *F. psychrophilum* within a population and exacerbating disease outbreaks.

Shedding of *F. psychrophilum* from infected fish into the water column is a primary factor driving horizontal transmission (13, 14). Madetoja et al. (14) showed that live rainbow trout shed up to ~10^6^ cells/fish/hour for up to 21 days. Notably, the same authors also showed that dead rainbow trout shed *F. psychrophilum* at even higher rates (e.g., up to ~10^8^ cells/fish/hour) and for a longer duration (e.g., up to 59 days longer) (14). Although these studies provided invaluable data related to *F. psychrophilum* shedding dynamics (e.g., time to shedding and shedding rate and duration) in rainbow trout, they represent the totality of knowledge on this subject. Thus, it remains to be determined if *F. psychrophilum* shedding dynamics in other BCWD-susceptible host species, such as Atlantic salmon and coho salmon, are similar.

In a similar context, whether *F. psychrophilum* shedding dynamics vary according to the *F. psychrophilum* multilocus sequence typing (MLST) variant causing the epizootic has yet to be determined. Multilocus sequencing typing of >1,500 *F*. *psychrophilum* isolates has revealed >260 different sequence types (STs) worldwide (https://pubmlst.org/organisms/flavobacterium-psychrophilum), some of which differ in host species association (15–17), virulence (18, 19), and prevalence in either hatchery-reared or wild/feral fish populations (15, 20). Notably, most *F. psychrophilum* STs in North America differ from those that have been reported from other continents (e.g., Asia, Europe, South America) (15, 17, 20–26), suggesting that the *F. psychrophilum* variant evaluated by Madetoja et al. (14) is likely distinct from many causing losses in the USA.

The detection and quantification of *F. psychrophilum* from water containing fish have been attempted via culture and immunofluorescence antibody technique (14, 27, 28), both of which are sensitive [e.g., detection limit of ~10^1^–10^2^ colony-forming units (cfu)/mL) but time consuming and vary in specificity. An alternative that is highly sensitive, specific, and well suited for *F. psychrophilum* quantification in water is quantitative PCR (qPCR). Strepparava et al. (29) developed an *F. psychrophilum* qPCR for this purpose, and although sensitive (detection limit of ~10^1^ gene copies) and specific, its quantification limit was reportedly high (e.g., ~10^3^ *F. psychrophilum* cells/mL). A more sensitive *F. psychrophilum*-specific qPCR exists (detection limit of ~10^0^ gene copies) (30), but in the published literature, this assay has not been used to quantify *F. psychrophilum* loads in water.

Toward improving our understanding of *F. psychrophilum* shedding dynamics in rainbow trout and, for the first time, Atlantic salmon and coho salmon, a series of experiments were devised to first optimize a previously developed *F. psychrophilum*-specific qPCR (30) for detection and quantification in water. Next, *in vivo* experiments were designed to elucidate *F. psychrophilum* shedding rates and durations in live and dead Atlantic salmon, coho salmon, and rainbow trout. Clarifying these aspects of BCWD ecology will offer insights into improved BCWD management strategies for multiple valuable salmonid species.

## MATERIALS AND METHODS

### Validation of modified method of Marancik and Wiens qPCR for detection of *Flavobacterium psychrophilum* DNA in water

#### Reaction mixture and cycling parameters

The *F. psychrophilum*-specific qPCR developed by Marancik and Wiens (30) was performed according to protocol with some modification. Briefly, each 15-µL reaction mixture contained 7.5 µL of TaqMan Environmental Master Mix 2.0, 0.67 µM of forward and reverse primers, 0.17 µM of TaqMan probe, 0.60 µL of VetMAX Xeno Internal Positive Control (IPC) Assay, and 1 µL of template, with nuclease-free water comprising the remainder. Reactions were run in MicroAmp Optical 96-Well Fast Reaction Plates (0.1 mL) covered with MicroAmp Optical Adhesive Film. A QuantStudio 3 real-time thermal cycler (Thermo Fisher Scientific) was used to amplify the 77-bp target amplicon according to the cycling program of Marancik and Wiens (30). All consumables were purchased through Thermo Fisher Scientific except the primers, which were obtained from Integrated DNA Technologies (IDT).

#### Preparation of standards

The qPCR target gene sequence was PCR amplified using previously extracted gDNA from *F. psychrophilum* isolate US53 (20) and the same primers used for qPCR. Briefly, a 50-µL reaction mixture was prepared with 25-µL 2X GoTaq Green Master Mix (Promega), 0.25 µM forward and reverse primers, 40 ng of US53 gDNA, with nuclease-free water comprising the remainder. A touchdown protocol consisting of initial denaturation for 2 min at 94°C followed by 30 cycles of 94°C for 1 min, 60°C for 1 min, 72°C for 1 min, and a final extension step at 72°C for 7 min was performed using an Eppendorf Mastercycler pro conventional thermal cycler (Thermo Fisher Scientific). The PCR product was run through a 1.5% agarose gel prepared with 1× SYBR Safe DNA gel stain (Thermo Fisher Scientific) for 40 min at 100 V, after which the gel was viewed under UV transillumination to confirm the presence of an appropriately sized band. The PCR product (57.1% guanine-cytosine content; molecular weight of 37,497.9 g mol^−1^) (30) was purified using the QIAquick PCR Purification Kit (Qiagen) and then quantified using a Qubit fluorometer and the broad range Quant-iT dsDNA Assay Kit (Thermo Fisher Scientific). Gene copy concentration was calculated using a previously published formula (31):


$$Gene\;copies/\mu L=\;\frac{\left(ngDNA\;\mu L^{-1}\right)\times \left(6.022\times 10^{23}\;copies\;mol^{-1}\right)}{\left(37,497.9\;g\;mol^{-1}\right)\times \left(1\times 10^{9}\;ng\;g^{-1}\right)}$$

Serial 10-fold dilutions of purified amplicon were made over nine orders of magnitude (e.g., 10^8^ copies/μL to 10° copies/μL) using 1X IDTE solution (pH 8.0; Integrated DNA Technologies) supplemented with 100 ng/µL tRNA (Yeast tRNA; Thermo Fisher Scientific). Two standard curve assays were performed, each on individual 96-well plates using eight replicate reactions of each dilution (i.e., qPCR standard); intra- and inter-assay variation was determined using mean quantification cycle (Cq), Cq standard deviation, and coefficient of variation (CV). Assay efficiency, slope, and the correlation coefficient (*R*^2^) of each assay were also calculated; efficiency estimates between 90% and 110% were considered acceptable (32, 33). These newly generated standards were used to determine *F. psychrophilum* DNA extraction efficiency, the limit of *F. psychrophilum* quantification and detection from spiked water (DNA extraction efficiency and limit of quantification and detection section), and to quantify *F. psychrophilum* loads in 50-mL water samples obtained during the *in vivo* shedding experiments (Determination of *Flavobacterium psychrophilum* shedding rate from live and dead fish via qPCR section).

### Optimization of water sampling method and qPCR for *Flavobacterium psychrophilum* quantification from water

#### Preparation of mock water samples containing *Flavobacterium psychrophilum*

*Flavobacterium psychrophilum* isolate US53 was revived from cryostock (maintained at −80°C) on *F. psychrophilum* medium A (FPM-A) (34), incubated for 48 hours at 15°C, visually inspected for purity, inoculated into 250 mL of analogous broth, and incubated with constant shaking (180 rpm) for 48 hours at 15°C. Bacteria were harvested via centrifugation (2,571 × *g*, 15 minutes) and resuspended in 50 mL of ultraviolet light-treated, sand-filtered well water (i.e., the same water supplying the shedding experimental aquaria in *In vivo* assessment of shedding dynamics in Atlantic salmon, coho salmon, and rainbow trout section), which was then serially diluted up to 100,000,000-fold in 10-fold increments to create nine total mock water samples. To quantify bacteria in the most concentrated mock water sample, a 1-mL aliquot was serially diluted 100,000,000-fold in 10-fold increments and plated on FPM-A in duplicate and then incubated for 7 days, after which final colony counts were performed. All mock water samples were brought to a final volume of 50 mL to replicate the water sampling volume used during the shedding experiment.

#### Bacterial DNA extraction from water

Each mock water sample (*n* = 9; Preparation of mock water samples containing *Flavobacterium psychrophilum* section) was vacuum filtered through a single sterile piece of Whatman qualitative filter paper (grade 4, 20–25 μm pore size, 70 mm in diameter; Millipore Sigma) that had been placed in a 70-mm diameter Büchner funnel. The filter paper was removed from the Büchner funnel and placed into a sterile Petri dish; sterile forceps were used to first fold the paper in half (the side receiving the bacterial suspension was facing inward) and then loosely roll it into a cylindrical shape. The rolled paper was placed inside a PowerBead Pro tube of the DNeasy PowerSoil Pro Kit (Qiagen) along with 20,000 copies of Xeno IPC to monitor inhibition; DNA was then extracted according to the manufacturers’ protocol, resulting in 50 µL of eluted DNA per dilution.

#### DNA extraction efficiency and limit of quantification and detection

The qPCR standards (Preparation of standards section) were used to simultaneously determine (i.e., on one 96-well plate) *F. psychrophilum* DNA extraction efficiency and the limit of detection (LOD) and quantification (LOQ). DNA extraction efficiency was measured as it is highly variable (e.g., 0.2%–108.9%) (35–40), thereby potentially affecting estimation of the target microorganism in a sample (e.g., *F. psychrophilum* in water) and, therefore, the LOD/LOQ (35).

*Flavobacterium psychrophilum* DNA extraction efficiency was calculated as the quotient of the mean qPCR-determined concentration of *F. psychrophilum* (in cells per milliliter) and the mean theoretical input (i.e., pre-DNA extraction) concentration of *F. psychrophilum* (41). The input concentration of *F. psychrophilum* was considered theoretical, given that the most concentrated mock water sample alone was quantified via plate counts (Preparation of mock water samples containing *Flavobacterium psychrophilum* section). The median of the mean *F. psychrophilum* DNA extraction efficiency values was used as the universal DNA extraction efficiency (35, 41); this number was used to apply a DNA extraction correction factor (DECF) to all qPCR-derived *F. psychrophilum* concentrations according to the following formula:


$$DECF=\;\frac{100}{Median\;\;of\;\;mean\;\;DNA\;\;extraction\;\;efficiency}$$

The LOD was determined by running 10 replicate reactions theoretically containing 100, 10, and 1 *F*. *psychrophilum* cell(s)/mL and defined as the lowest mean qPCR-derived concentration of *F. psychrophilum* that could be detected in ≥95% of qPCR replicate reactions; this concentration was multiplied by the DECF to yield the LOD (41).

The LOQ was determined by running triplicate reactions of eight qPCR standards (10^8^–10^1^ gene copies/reaction), triplicate reactions of six *F. psychrophilum* dilutions (1.00 × 10^8^ cells/mL – 1.00 × 10^3^ cells/mL), and 10 replicate reactions of each remaining dilution (e.g., 1 × 10^2^ cells/mL to 1 cell/mL) and defined as the lowest mean qPCR-derived concentration of *F. psychrophilum* with a CV <25%; this concentration was multiplied by the DECF to yield the LOQ (41).

### *In vivo* assessment of shedding dynamics in Atlantic salmon, coho salmon, and rainbow trout

#### *Flavobacterium psychrophilum* isolate selection

Three *F. psychrophilum* variants, including US19 (ST13, Clonal Complex [CC]-ST9) (20), US62 (ST277, CC-ST232) (15), and US87 (ST275, CC-ST10) (15), were selected for this study. Each CC is present across a wide geographic range, whereby CC-ST232 has been detected in two continents (Europe and North America) (15, 24), and CC-ST9 and CC-ST10 have both been detected in four continents (e.g., Asia, Europe, North America, and South America) (15, 21–24). Moreover, each CC has been recovered almost exclusively from one salmonid species, including Atlantic salmon (CC-ST232), coho salmon (CC-ST9), and rainbow trout (CC-ST10).

#### Origin of fish for shedding experiment

Embryonated Atlantic salmon and rainbow trout eggs were sourced from a commercial egg distributor, while embryonated coho salmon eggs were procured from Platte River State Fish Hatchery. Coordination occurred so that all eggs from the three species arrived at the Michigan State University–University Research Containment Facility on the same day. In brief and upon receipt, eggs were disinfected with 100-ppm iodophor solution (pH 7.30) for 10 minutes before being placed in a vertical incubator supplied with UV-treated, sand-filtered well water maintained at 12°C ± 1°C until hatching. Sac-fry were then moved to aerated flow-through tanks (40 L; 12°C ± 1°C) and, once exogenous feeding commenced, were given a continuous supply of appropriately sized commercial trout food (Skretting, the Netherlands) via an automatic feeder. After 8 weeks, fish were hand fed twice daily, and the water volume in the tanks was increased (400 L; 12°C ± 1°C). Tanks were cleaned and siphoned daily to remove waste and any uneaten food. Before the challenge experiment, a subset of fish from each species was cultured to screen for bacterial infections (16), including those caused by *F. psychrophilum*, and confirmed to be bacterial infection free.

#### *Flavobacterium psychrophilum* inoculum preparation for shedding experiment

*Flavobacterium psychrophilum* variants US19, US62, and US87 were revived from cryostock and verified to be pure cultures as detailed in Preparation of mock water samples containing *Flavobacterium psychrophilum* section. Each *F. psychrophilum* variant was inoculated into 250 mL of FPM-A broth and incubated with constant shaking (180 rpm) for 48 hours at 15°C. Bacteria were harvested via centrifugation (2,571 × *g*, 15 minutes) and adjusted to an optical density at 600 nm (OD_600_) corresponding to 2.0 × 10^8^ cfu/mL using sterile 0.65% saline. Concentrations of each *F. psychrophilum* variant were determined as detailed in Preparation of mock water samples containing *Flavobacterium psychrophilum* section.

#### Intramuscular challenge of fish

Each *F. psychrophilum* variant was inoculated into the salmonid species it is associated with, according to MLST. Thus, US19 was inoculated into coho salmon, whereas US62 and US87 were inoculated into Atlantic salmon and rainbow trout, respectively.

Atlantic salmon (*n =* 10; 8 months old; mean weight 18.1 g), coho salmon (*n* = 10; 8 months old; mean weight 20.5 g), and rainbow trout (*n =* 10; 8 months old; mean weight 25.1 g) were anesthetized in sodium bicarbonate-buffered (200 mg/L) tricaine methanesulfonate (MS-222; Syndel) at a concentration of 100 mg/L. Then, fish were intramuscularly injected at the base of the dorsal fin with a 50-µL volume of *F. psychrophilum*, equating to a dose of 10^5^ cfu/g, and then placed into aerated flow-through glass experimental aquaria (37.85 L; *n* = 2 aquaria per species, *n =* 5 fish per aquarium) supplied with ultraviolet light-treated, sand-filtered well water (12°C ± 1°C). Control fish (*n* = 5 per species, in duplicate aquaria) were treated identically except they were intramuscularly injected with an equal volume of 0.65% saline. The challenge experiment was conducted in accordance with the MSU-Institutional Animal Care and Use Committee (AUF:201900312).

#### Sampling of water containing live and dead fish

All live fish in a replicate experimental aquarium (i.e., main aquarium) were net transferred to a clean non-flow-through glass aquarium (i.e., shedding aquarium; 9.46 L) containing 3,000 mL of fresh, ultraviolet light-treated, sand-filtered well water (12°C ± 1°C) with constant aeration. The shedding aquarium was placed inside a larger, opaque, plastic aquarium that had flow-through water to maintain a water temperature of 12°C ± 1°C in the shedding aquarium; the plastic aquarium also had an opaque cover to minimize light-induced stress. After 1 hour, all fish were removed from the shedding aquarium and transferred back to the main aquarium. A 50-mL water sample was collected using a sterile 50-mL conical tube and then processed immediately as detailed in Bacterial DNA extraction from water section. Water sampling of live fish (including negative control fish) occurred on every other day for the first week, twice per week during the second and third weeks, and then once a week until the end of the experiment (i.e., 4 weeks without detection of *F. psychrophilum*).

Up to two dead fish per replicate aquarium (i.e., ≤4 dead fish per species) were net transferred into a new flow-through glass aquarium (37.85 L, *n* = 1 dead fish per aquarium) supplied with ultraviolet light-treated, sand-filtered well water (12°C ± 1°C). After 1, 3, 5, 7, 14, 63, and 98 days post death (PD), fish were transferred to individual shedding aquaria and treated/sampled identically to the live fish. Fish that died that were not used for determining *F. psychrophilum* shedding rates were necropsied and clinically examined, and multiple tissues (e.g., external ulcers and kidney) were bacteriologically analyzed for *F. psychrophilum* on FPM-A. To disinfect shedding aquaria between samplings, aquaria were completely immersed in a 10% (vol/vol) bleach solution for ≥10 minutes, rinsed thoroughly with pathogen-free water, and then air dried.

#### Determination of *Flavobacterium psychrophilum* shedding rate from live and dead fish via qPCR

*Flavobacterium psychrophilum* shedding rates were determined via qPCR (Reaction mixture and cycling parameters section). Briefly, each 96-well qPCR plate consisted of triplicate reactions of eight qPCR standards (10^8^ gene copies to 10^1^ gene copies), duplicate reactions of template DNA, triplicate reactions of no-template control (e.g., sterile, nuclease-free water), and triplicate reactions of IPC amplification control (e.g., 1,000 copies of Xeno IPC). The shedding rate of *F. psychrophilum* from infected fish is reported as *F. psychrophilum* cells shed per fish per hour in 1 mL of water (i.e., *F. psychrophilum* cells/fish/hour) (14) and was calculated using the following formula:


$$F.\;psychrophilum\;\;cells/fish/hour=\;\frac{Mean\;\;qPCR\;\;gene\;\;copies\;\times \;DECF\;\times \;3000\;\;mL}{Number\;\;of\;\;fish\;\;in\;\;aquarium}$$

## RESULTS

### qPCR standards

All qPCR standards, which spanned nine orders of magnitude (10^8^ gene copies/reaction to 1 gene copy/reaction), successfully amplified. Linear regression of C_q_ values vs the log_10_ target gene demonstrated acceptable correlation (*R*^2^ = 0.999 ± .001) with a slope and efficiency of −3.23 ± 0.01 and 104.01% ± 0.53%, respectively. The assay was repeatable within and between runs, as CV values were <2.5% within the linear range (10^8^ gene copies/reaction to 1 gene copy/reaction) of the standard curve (Table 1). Consequently and in accordance with Standish et al. (31), these standards were used in all subsequent qPCR assays.

**TABLE 1:** Repeatability of *Flavobacterium psychrophilum* Marancik and Wiens qPCR assay^*a*^

Gene copies	C_q_ mean	C_q_ SD	C_q_ CV (%)
Intra-assay			
100,000,000	11.34	0.12	1.06
10,000,000	14.63	0.09	0.65
1,000,000	17.83	0.09	0.48
100,000	21.78	0.11	0.52
10,000	24.61	0.12	0.48
1,000	27.60	0.11	0.40
100	30.76	0.16	0.51
10	34.05	0.36	1.06
1	36.96	0.86	2.32
Inter-assay			
100,000,000	11.22	0.12	1.09
10,000,000	14.65	0.17	1.17
1,000,000	17.79	0.10	0.57
100,000	21.58	0.13	0.61
10,000	24.49	0.20	0.83
1,000	27.58	0.12	0.42
100	30.69	0.20	0.65
10	34.01	0.31	0.90
1	37.05	0.83	2.25

^*a*^ Mean, standard deviation, and coefficient of variation (CV) of the cycle threshold (C_q_) values are shown. To determine intra-assay variation, the target gene was serially diluted in 10-fold increments over nine orders of magnitude (i.e., 10^8^ gene copies/reaction to 1 gene copy/reaction), and eight replicate reactions of each dilution were PCR amplified and quantified. Inter-assay variation was determined by repeating the qPCR run on a separate plate using an identical number of replicate reactions. The assay was repeatable within and between runs, as CV percentages were <2.5 across the linear range (31).

### *Flavobacterium psychrophilum* DNA extraction efficiency and qPCR limit of detection and quantification

Mean *F. psychrophilum* concentrations of the nine mock water samples ranged from 1.00 × 10^8^ to 1.00 × 10° cells/mL and were compared to the mean qPCR-determined concentrations (Table 2). Mean DNA extraction efficiency ranged from 3.63% to 99.70%, with a median of 14.03% (Table 2); therefore, the DECF applied to all qPCR-derived concentrations was 7.128 (100/14.03) (35).

**TABLE 2:** Evaluation of *Flavobacterium psychrophilum* DNA extraction procedure from water using qPCR^*c*^^,^^*d*^

Theoretical concentration of *F. psychrophilum*	qPCR-derived concentration of *F. psychrophilum*						*F. psychrophilum* DNA extraction efficiency (%)^*b*^
	Mean	SD	Signal ratio^*a*^	CV (%)	C_q_ IPC (SD)	IPC CV (%)	
1.00 × 10^8^	9.97 × 10^7^	5.21 × 10^6^	3/3	5.23	–	–	99.70
1.00 × 10^7^	7.85 × 10^6^	7.08 × 10^4^	3/3	0.90	35.29 (0.32)	0.91	78.49
1.00 × 10^6^	3.19 × 10^5^	2.82 × 10^4^	3/3	8.83	31.08 (0.26)	0.82	31.91
1.00 × 10^5^	8.87 × 10^3^	4.35 × 10^2^	3/3	4.90	30.12 (0.21)	0.70	8.87
1.00 × 10^4^	6.73 × 10^2^	3.30 × 10^1^	3/3	4.90	31.17 (0.15)	0.49	6.73
1.00 × 10^3^	3.63 × 10^1^	6.07 × 10^0^	3/3	16.74	32.31 (0.11)	0.36	3.63
1.00 × 10^2^	4.34 × 10^0^	9.63 × 10^−1^	10/10	22.19	31.56 (0.37)	1.17	4.34
1.00 × 10^1^	1.92 × 10^0^	9.95 × 10^−1^	4/10	51.81	31.04 (0.24)	0.76	19.20
1.00 × 10^0^	–	–	0/10	–	32.12 (0.36)	1.11	–
Median							14.03

^*a*^ Calculated as the quotient of the mean qPCR-derived concentration of *F. psychrophilum* and the mean theoretical concentration of *F. psychrophilum* cells, multiplied by 100.

^*b*^ Number of amplified replicate reactions/total number of replicate reactions.

^*c*^ SD, standard deviation; CV, coefficient of variation; IPC, internal positive control; C_q_, cycle threshold.

^*d*^ "–" represents an undeterminable value.

The assay LOD and LOQ were calculated as 30.94 ± 6.84 cells/mL, whereby the DECF (7.128) was multiplied by the lowest mean qPCR-derived *F. psychrophilum* concentration meeting the qualifications for the LOD and LOQ (e.g., both 4.34 ± 0.96 cells/mL; Table 2). Additionally, amplification was achieved in 40% of the replicates at the next lowest concentration, which corresponded to 13.68 ± 7.09 cells/mL after correction for DNA extraction efficiency (1.92 ± 0.99 cells/mL × 7.128).

The IPC DNA, which was added at the beginning of the DNA extraction process, successfully amplified in all replicate reactions of all but the most concentrated mock water sample (Table 2), which according to the VetMAX Xeno user guide was most likely caused by high target concentration. Nonetheless, of the eight mock water samples that had successful IPC amplification, C_q_ values ranged from 30.12 to 35.29, and all replicates were highly consistent (e.g., CV values ranged from 0.36% to 1.17%; Table 2).

### *Flavobacterium psychrophilum* shedding dynamics in three salmonid species

#### Shedding rates of *Flavobacterium psychrophilum* from live fish

**Fig 1:**
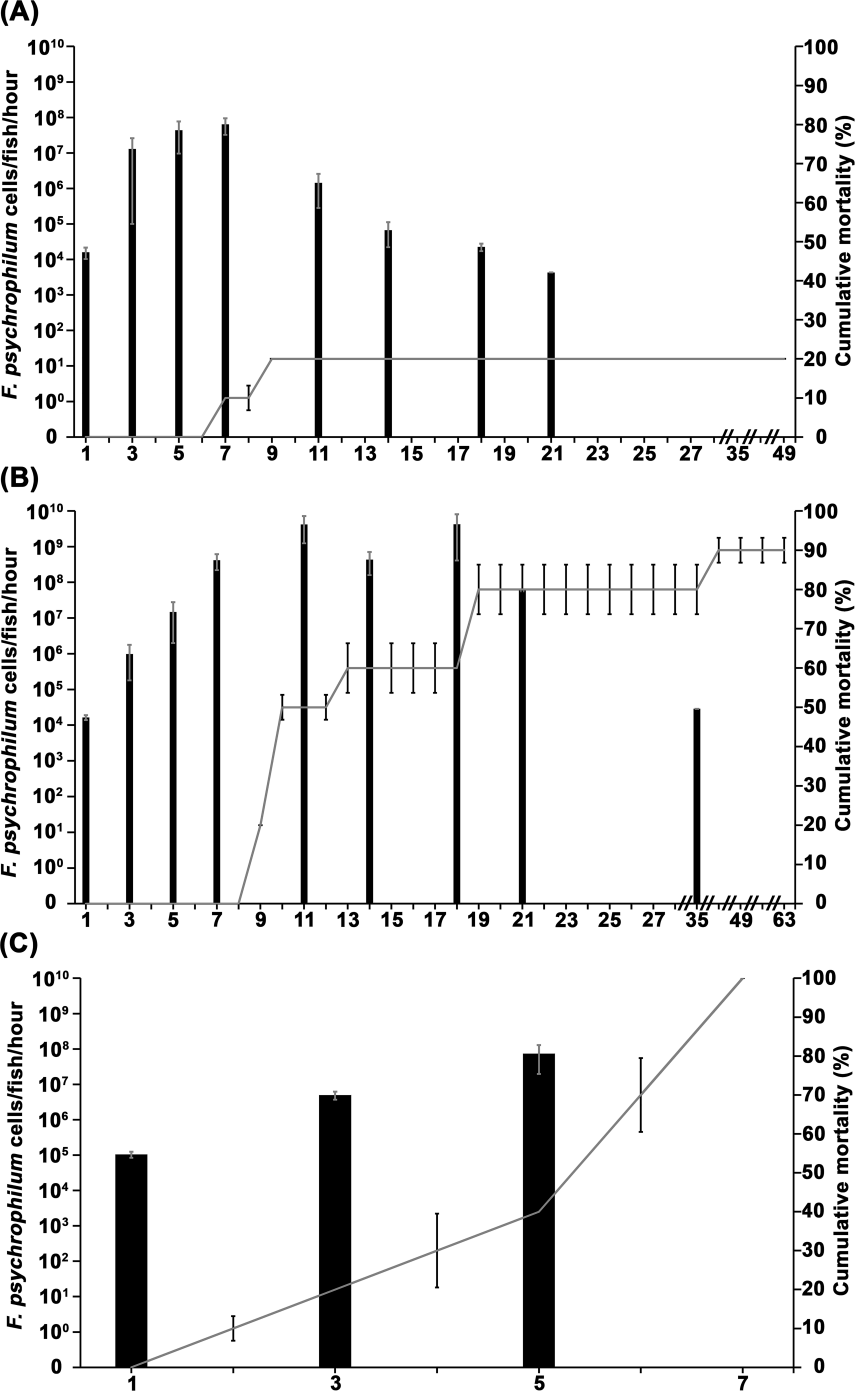
Mean *Flavobacterium psychrophilum* shedding rates (black bars with standard deviation) from and cumulative percent mortality (gray lines with standard error) of live (A) Atlantic salmon (*Salmo salar*), (B) rainbow trout (*Oncorhynchus mykiss*), and (C) coho salmon (*O. kisutch*). // indicates 6-day gap in time.

Following intramuscular injection of 10^5^ cfu/g of *F. psychrophilum*, all tested species (e.g., Atlantic salmon, coho salmon, and rainbow trout) shed *F. psychrophilum* into the water over multiple days and/or prior to the occurrence of any mortality (Fig. 1A through C).

Atlantic salmon shed 1.59 × 10^4^ cells/fish/hour 1 day after inoculation; this shedding rate increased to 4.34 × 10^7^ cells/fish/hour (i.e., >2,700-fold increase) by day 5, which was 2 days prior to the first mortality (Fig. 1A). Atlantic salmon shed the most *F. psychrophilum* (e.g., 6.37 × 10^7^ cells/fish/hour) on day 7 (i.e., the day of the first mortality; Fig. 1A), with shedding rates decreasing thereafter on each subsequent sampling day (e.g., from 1.43 × 10^6^ cells/fish/hour on day 11 to 4.30 × 10^3^ cells/fish/hour on day 21; Fig. 1A). Cumulative mortality in Atlantic salmon peaked at 20% on day 9. Surviving Atlantic salmon were euthanized on day 49 (i.e., 4 weeks past the last detection of *F. psychrophilum*; Fig. 1A), at which time *F. psychrophilum* infection status was examined (Infection status in salmonids challenged with *Flavobacterium psychrophilum* section).

Live rainbow trout began shedding *F. psychrophilum* into the water at a rate of 1.65 × 10^4^ cells/fish/hour 1 day after inoculation; this shedding rate increased to 4.16 × 10^8^ cells/fish/hour (i.e., >25,000-fold increase) by day 7, which was 2 days prior to the first mortality (Fig. 1B). Rainbow trout shedding rates then increased to 4.18 × 10^9^ cells/fish/hour on day 11 (50% cumulative mortality) and decreased to 4.32 × 10^8^ cells/fish/hour on day 14 (60% cumulative mortality) before increasing to 4.23 × 10^9^ cells/fish/hour on day 18 (60% cumulative mortality; Fig. 1B). On day 19, cumulative mortality increased to 80% and remained constant through day 35; during this time, rainbow trout shedding rates decreased from 5.98 × 10^7^ cells/fish/hour (day 21) to 2.86 × 10^4^ cells/fish/hour (day 35), after which *F. psychrophilum* was not detected in the water (Fig. 1B). Cumulative mortality in rainbow trout reached its peak (e.g., 90%) on day 42, and the experiment ended on day 63 (Fig. 1B).

Live coho salmon began shedding *F. psychrophilum* into the water at a rate of 1.04 × 10^5^ cells/fish/hour 1 day after inoculation. Mortality began quickly in this species (e.g., on day 2) and rapidly reached 100% by day 7. The highest detected *F. psychrophilum* shedding rate of live coho salmon (e.g., 7.39 × 10^7^ cells/fish/hour) occurred on day 5 (Fig. 1C).

Control fish did not shed *F. psychrophilum* nor did they experience any mortality (data not shown). Likewise, inhibition of gene target amplification was not observed among water samples originating from the negative control or *F. psychrophilum*-exposed fish, as evidenced by all IPC C_q_ values falling within 28–34.

#### Shedding rates of *Flavobacterium psychrophilum* from dead fish

**Fig 2:**
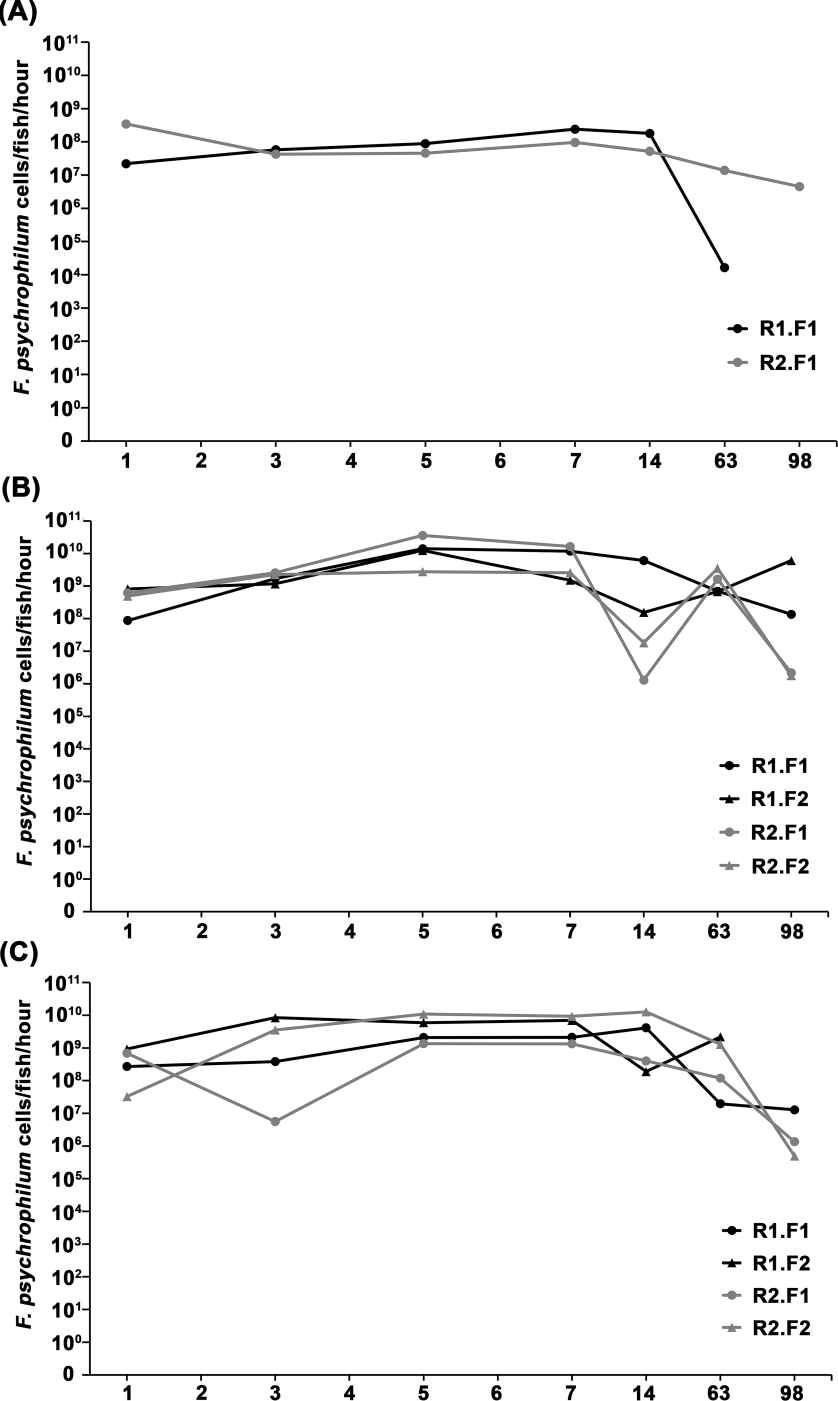
*Flavobacterium psychrophilum* shedding rates of dead individual (**A**) Atlantic salmon (*Salmo salar*), (**B**) rainbow trout (*Oncorhynchus mykiss*), and (**C**) coho salmon (*O. kisutch*). Legend explanation: ≤2 fish (**F**) per replicate (**R**) aquarium were maintained. Therefore, in the legend and as an example, R1.F1 corresponds to replicate one, fish one.

**Fig 3:**
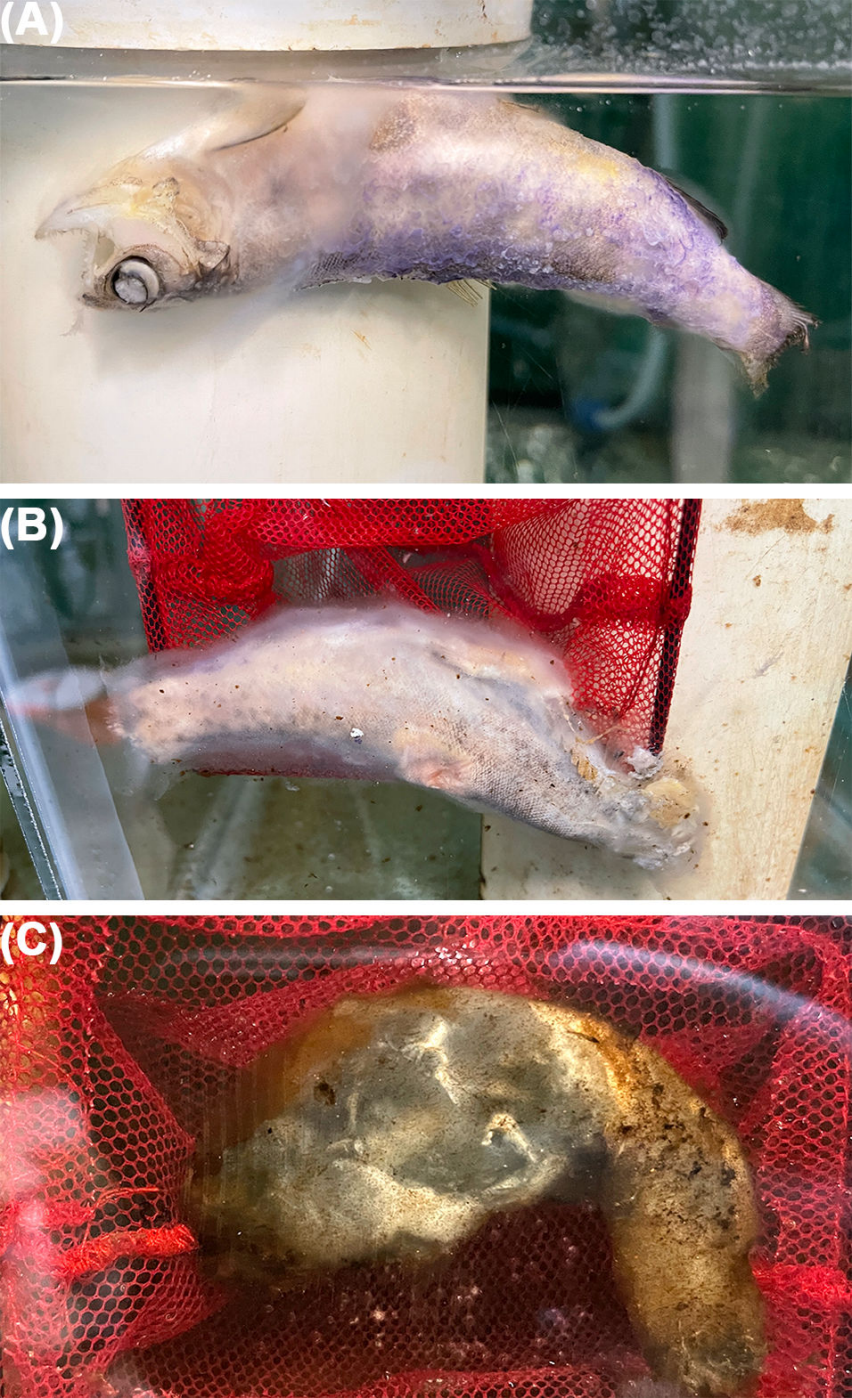
Representative images of dead fish used in *Flavobacterium psychrophilum* shedding experiment. (**A**) Coho salmon (*Oncorhynchus kisutch*) 12 days post death. (**B**) Rainbow trout (*O. mykiss*) 25 days post death. (**C**) Coho salmon 98 days post death. Note the yellowish discoloration present on fish.

Atlantic salmon, coho salmon, and rainbow trout shed *F. psychrophilum* into the water from 1 day PD to the end of the 98-day experiment (Fig. 2A through C). Throughout the experiment, fish underwent post-mortem decomposition (Fig. 3A through C). Because of extensive decomposition after day 98, sampling ceased.

Two Atlantic salmon died during the experiment, one from each replicate aquarium (Fig. 2A). Initially (i.e., 1 day PD), *F. psychrophilum* shedding rates differed by ~15-fold, whereby one fish shed 2.20 × 10^7^ cells/fish/hour and the other shed 3.45 × 10^8^ cells/fish/hour (mean of 1.8 ± 1.6 × 10^8^ cells/fish/hour; Fig. 2A; Table 3); however, shedding rates became more consistent between 3 and 14 days PD (e.g., differed by 1–3-fold). Over the next 12 weeks, the shedding rate of one Atlantic salmon decreased to 1.63 × 10^4^ cells/fish/hour and then *F. psychrophilum* became undetectable, whereas the shedding rate of the other Atlantic salmon decreased but was still detectable (e.g., 4.51 × 10^6^ cells/fish/hour; Fig. 2A) 98 days PD.

**TABLE 3:** Mean *Flavobacterium psychrophilum* shedding rates (cells/fish/hour) ± standard deviation of dead Atlantic salmon, coho salmon, and rainbow trout on each sampling day^*a*^

	Host species					
Sampling day	*N*	Atlantic salmon	*N*	Coho salmon	*N*	Rainbow trout
1	2	1.8 ± 1.6 × 10^8^	4	4.8 ± 3.5 × 10^8^	4	5.0 ± 2.7 × 10^8^
3	2	5.0 ± 0.8 × 10^7^	4	3.1 ± 3.4 × 10^9^	4	1.9 ± 0.5 × 10^9^
5	2	6.7 ± 2.1 × 10^7^	4	5.0 ± 3.8 × 10^9^	4	1.6 ± 1.2 × 10^10^
7	2	1.7 ± 0.7 × 10^8^	4	4.9 ± 3.3 × 10^9^	4	8.1 ± 6.3 × 10^9^
14	2	1.2 ± 0.6 × 10^8^	4	4.4 ± 5.1 × 10^9^	4	1.6 ± 2.6 × 10^9^
63	2	6.9 ± 6.9 × 10^6^	4	9.1 ± 8.9 × 10^8^	4	1.6 ± 1.2 × 10^9^
98	1	4.5 ± 0.0 × 10^6^	3	4.9 ± 5.6 × 10^6^	4	1.6 ± 2.6 × 10^9^

^*a*^ Number of fish sampled (*N*) is to the left of the host species name.

Of the 10 rainbow trout, 9 died during the experiment; therefore, individual *F. psychrophilum* shedding rates of two rainbow trout per replicate aquarium were measured (Fig. 2B). Initially (i.e., 1 day PD), most (i.e., 3/4) rainbow trout were shedding ~10^8^ cells/fish/hour (range of 4.91–8.20 × 10^8^ cells/fish/hour), whereas one rainbow trout was shedding 8.73 × 10^7^ cells/fish/hour (overall mean of 5.0 ± 2.7 × 10^8^ cells/fish/hour; Fig. 2B; Table 3). By 5 days PD, all rainbow trout shed at substantially higher rates, whereby individual rainbow trout were shedding 0.28–3.59 × 10^10^ cells/fish/hour (mean of 1.6 ± 1.2 × 10^10^ cells/fish/hour; Fig. 2B; Table 3). Between 7 and 14 days PD, rainbow trout shedding rates decreased ~1–12,000-fold, and the shedding rate of one rainbow trout continued to decrease through the end of the experiment (final shedding rate of 1.36 × 10^8^ cells/fish/hour; Fig. 2B). For the other three rainbow trout, shedding rates increased ~4–1,200-fold (e.g., to 0.68–3.5 × 10^9^ cells/fish/hour) by 63 days PD. At the end of the 98-day experiment, these three rainbow trout were shedding 1.77 × 10^6^ to 6.09 × 10^9^ cells/fish/hour (mean of 1.6 ± 1.2 × 10^9^ cells/fish/hour; Fig. 2B; Table 3).

All 10 coho salmon died during the experiment; therefore, individual *F. psychrophilum* shedding rates of two coho salmon per replicate aquarium were measured (Fig. 2C). Initially (i.e., 1 day PD), most (i.e., 3/4) coho salmon were shedding ~10^8^ cells/fish/hour (range of 2.71–9.26 × 10^8^ cells/fish/hour), whereas one coho salmon was shedding 3.24 × 10^7^ cells/fish/hour (overall mean of 4.8 ± 3.5 × 10^8^ cells/fish/hour; Fig. 2C; Table 3). By 3 days PD, shedding increased by ~1–100-fold for most (i.e., 3/4) coho salmon, whereby individuals were shedding between 0.38 and 8.45 × 10^9^ cells/fish/hour (Fig. 2C). In contrast, *F. psychrophilum* shedding rate decreased by ~100-fold (e.g., to 5.59 × 10^6^ cells/fish/hour) for the other coho salmon. By 5 days PD, shedding rates had increased for all coho salmon (range of 0.21 × 10^10^ cells/fish/hour; mean of 5.0 ± 3.8 × 10^9^ cells/fish/hour; Fig. 2C; Table 3) and continued to increase for 2/4 coho salmon through 7 days PD (range of 2.12–6.94 × 10^9^ cells/fish/hour). In the other two coho salmon, a decrease in shedding rates was appreciated (range of 1.34–9.28 × 10^9^ cells/fish/hour; Fig. 2C). Over the next 13 weeks (i.e., through the end of the 98-day experiment), shedding rates generally decreased, and most (i.e., 3/4) coho salmon were still shedding *F. psychrophilum* (range of 4.95 × 10^5^ to 1.28 × 10^7^ cells/fish/hour; mean of 1.6 ± 2.6 × 10^9^ cells/fish/hour; Fig. 2C; Table 3).

### Infection status in salmonids challenged with *Flavobacterium psychrophilum*

*Flavobacterium psychrophilum* was recovered in a pure form and as perfuse lawns (i.e., colony-forming units were too numerous to count) from external lesions (e.g., muscle ulcerations) and the kidneys of all dead Atlantic salmon, coho salmon, and rainbow trout. *Flavobacterium psychrophilum* was not recovered from the kidneys of any surviving Atlantic salmon (*n =* 8 fish; 49 days post inoculation) or rainbow trout (*n* = 1 fish; 63 days post inoculation). However, *F. psychrophilum* was recovered in a pure form and as a perfuse lawn from the eye of the only surviving rainbow trout. Because all coho salmon died after 7 days, no survivors could be cultured.

## DISCUSSION

For the first time, data on *F. psychrophilum* shedding dynamics (e.g., time to shedding, shedding rate, and duration in live and dead fish) in Atlantic salmon and coho salmon, which are two of the most BCWD-susceptible salmonid species (3, 6, 7), have been elucidated. Addressing this knowledge gap was essential, as several studies showed that BCWD epizootics in Atlantic salmon and coho salmon are instigated by phenotypically distinct *F. psychrophilum* variants (14, 19, 42). Moreover, these *F. psychrophilum* variants differ from those affecting rainbow trout (15, 21, 24), the sole species our entire understanding of *F. psychrophilum* shedding dynamics is based upon. Although some commonalities in shedding dynamics were observed among Atlantic salmon, coho salmon, and rainbow trout, multiple differences were also apparent.

Dead Atlantic salmon, rainbow trout, and coho salmon were found to shed *F. psychrophilum* cells at rates up to 5.4-, 8.5-, and 171.8-fold greater than their living counterparts and did so for extended periods (e.g., up to 77, 63, and 93 days longer). This is the first time that these comparisons have been made among Atlantic salmon and coho salmon, demonstrating that these species, in addition to rainbow trout, are efficient *F. psychrophilum* shedders. Madetoja et al. (14) also found dead rainbow trout shed at higher (e.g., up to ~100-fold) rates and for a longer duration (e.g., 59 days longer) than living rainbow trout. The difference in shedding rate among live and dead rainbow trout observed by Madetoja et al. (14) is substantially greater than the 8.5-fold difference found herein for rainbow trout, while differences in shedding duration were similar (59 vs 63 days) (14). Taken together with the findings in Atlantic salmon and coho salmon, results suggest that some *F. psychrophilum* variants may be more efficiently shed by dead fish than others. Nevertheless, dead *F. psychrophilum*-infected fish clearly pose a significant risk for disease perpetuation within fish farms and hatcheries, underscoring the importance of implementing management strategies that aim to remove dead fish from rearing units quickly. Like *F. psychrophilum*, higher shedding rates in dead vs live salmonids have been noted in other fish pathogenic *Flavobacterium* species (e.g., *F. columnare*, a cause of columnaris disease) (43, 44) as well as *Aeromonas salmonicida* subspecies *salmonicida* (e.g., the cause of furunculosis) (45), highlighting dead fish as potential transmission risks for multiple bacterial fish pathogens.

Despite some similarities, multiple differences in *F. psychrophilum* shedding dynamics by host species and variant were also observed. For example, on day 11, live rainbow trout were shedding 2,923-fold more *F. psychrophilum* cells per hour compared to live Atlantic salmon. This difference continued to increase through day 21, which was the last day of *F. psychrophilum* detection among Atlantic salmon. Meanwhile (i.e., on day 21), *F. psychrophilum* shedding rates remained high among rainbow trout (e.g., 5.98 × 10^7^ cells/fish/hour), and this species continued to shed for another 2 weeks. Adding a further layer of complexity, *F. psychrophilum* shedding rates among live Atlantic salmon and rainbow trout tended to peak on and/or near days with mortality and could possibly suggest that risk of transmission may vary by *F. psychrophilum* variant. For example, Atlantic salmon infected with US62 (ST277, in CC-ST232) died over 3 days, whereas rainbow trout infected with US87 (ST275) mostly died over 11 days (i.e., a greater than threefold longer period). Madetoja et al. (14) infected rainbow trout with a different *F. psychrophilum* variant and observed that fish died over a 4-day period. Therefore, it appears that some *F. psychrophilum* variants pose a greater transmission risk among live fish compared to others.

The observed differences in shedding dynamics across *F. psychrophilum* variants are likely influenced by pathogen and/or host factors. In this study, US87, identified in reference (46) as molecular serotype 2 (nearly equivalent to conventional serotype Th) (47, 48), showed a protracted shedding period in rainbow trout and persisted systemically in a survivor (e.g., for at least 63 days), suggesting that the rainbow trout immune system may struggle in eliminating this variant. In the context of a fish farm or hatchery, this outcome could affect *F. psychrophilum* transmission dynamics and increase the number of subsequent infections (i.e., the basic reproduction number, R_0_) (49). US62 also exhibited prolonged shedding herein, albeit to a lesser extent than US87, but appeared to be successfully cleared by Atlantic salmon at the end of the experiment. This *F. psychrophilum* variant belonged to molecular serotype 1 (nearly equivalent to conventional serotype Fd) (46–48), which is less common in Atlantic salmon (4) and thus possibly less capable of circumventing this species’ immune response, possibly helping to explain the shorter duration of shedding and lack of recovery from survivors. US19 was highly virulent herein, despite using an identical dose, and caused mortality before a rigorous adaptive immune response could be mounted and additional shedding data collected. Whether the shedding dynamics of these variants are representative of other *F. psychrophilum* variants affecting Atlantic salmon, coho salmon, and rainbow trout should be further investigated.

In addition to elucidating several aspects of *F. psychrophilum* shedding dynamics in three salmonid species, this study also built upon the previous work of Marancik and Wiens (30) to optimize their qPCR assay for detection and quantification of *F. psychrophilum* in water. Originally, Marancik and Wiens (30) described this assay for the detection/quantification of *F. psychrophilum* in spleen tissue, with an LOD of 3.1 genome units per reaction and LOQ of ~486 colony-forming units. Herein, this assay was further optimized to quantify *F. psychrophilum* from filtered water, with an LOD/LOQ of 30.94 ± 6.84 cells/mL. The sensitivity of the assay could potentially be improved by filtering a larger water volume and/or by increasing sample volume per reaction. Indeed, even a 50-mL water volume containing 10^8^ *F. psychrophilum* cells/mL filtered quickly (i.e., no indication of filter fouling). Strepparava et al. (29) also designed a *F. psychrophilum*-specific qPCR to quantify *F. psychrophilum* from water samples, and although the LOD was similar (e.g., 66 cells/mL), the LOQ was >100-fold higher (e.g., 3,300 cells/mL). Moreover and in the hands of the study authors and at least two other laboratories, specificity issues were observed with this qPCR assay (unpublished data), and so, alternatives were sought. An additional improvement for the qPCR assay herein was the addition of an internal positive control, which allowed for monitoring of PCR-inhibition, a known source of “false negatives” with molecular assays (50). Moving forward, this qPCR assay will be instrumental to future studies assessing *F. psychrophilum* shedding dynamics and could have application to *F. psychrophilum* detection in environmental field settings.

Prior to this study, knowledge of *F. psychrophilum* shedding dynamics was limited only to rainbow trout and a single *F. psychrophilum* variant. Herein, dead Atlantic salmon, coho salmon, and rainbow trout were shown to shed *F. psychrophilum* at higher rates than their living counterparts and for at least several months, thereby potentially posing great transmission risk. Therefore and with the insight provided by a recent *F. psychrophilum* transmission model (51), it remains critical for personnel raising fish to remove dead, *F. psychrophilum*-infected fish from rearing units quickly and frequently. Given that *F. psychrophilum* is now well recognized as a genetically diverse pathogen (15, 17, 23, 24) of varying phenotypes that can affect salmonid hosts differentially (46) in conjunction with the differential shedding results by variant/host species herein, future studies should evaluate the risk of transmission to multiple salmonid species.

## Data Availability

The data that support the findings of this study are available from the corresponding author upon reasonable request.

## References

[1] Loch TP, Faisal M. 2017. *Flavobacterium* spp, p 211–232. In Woo PTK, Cipriano RC (ed), Fish viruses and bacteria: pathobiology and protection. CABI.

[2] Starliper CE. 2011. Bacterial coldwater disease of fishes caused by Flavobacterium psychrophilum. J Adv Res 2:97–108. doi:10.1016/j.jare.2010.04.001

[3] Holt RA. 1987. PhD thesis. Cytophaga psychrophila, the causative agent of bacterial cold water disease in Salmonid fish. Oregon State University, Corvallis, OR.

[4] Avendaño-Herrera R, Tapia-Cammas D, Duchaud E, Irgang R. 2020. Serological diversity in Flavobacterium psychrophilum: a critical update using isolates retrieved from Chilean salmon farms. J Fish Dis 43:877–888. doi:10.1111/jfd.13199 32567047

[5] Macchia V, Inami M, Ramstad A, Grammes F, Reeve A, Moen T, Torgersen JS, Adams A, Desbois AP, Hoare R. 2022. Immersion challenge model for Flavobacterium psychrophilum infection of Atlantic salmon (Salmo salar L.) fry. J Fish Dis 45:1781–1788. doi:10.1111/jfd.13699 36223485 PMC9804593

[6] Nilsen H, Johansen R, Colquhoun DJ, Kaada I, Bottolfsen K, Vågnes Ø, Olsen AB. 2011. Flavobacterium psychrophilum associated septicaemia and necrotic myositis in Atlantic salmon Salmo salar: a case report. Dis Aquat Organ 97:37–46. doi:10.3354/dao02390 22235593

[7] Barnes ME, Brown ML. 2011. A review of Flavobacterium psychrophilum biology, clinical signs, and bacterial coldwater disease prevention and treatment. TOFISHSJ 4:40–48. doi:10.2174/1874401X01104010040

[8] Wood JW. 1974. Diseases of Pacific salmon, their prevention and treatment. 2nd ed. Washington Department of Fisheries, Olympia.

[9] Brown LL, Cox WT, Levine RP. 1997. Evidence that the causal agent of bacterial Coldwater disease Flavobacterium psychrophilum is transmitted within salmonid eggs. Dis Aquat Org. 29:213–218. doi:10.3354/dao029213

[10] Ekman E, Börjeson H, Johansson N. 1999. Flavobacterium psychrophilum in baltic salmon Salmo salar brood fish and their offspring. Dis Aquat Organ 37:159–163. doi:10.3354/dao037159 10546045

[11] Kumagai A, Yamaoka S, Takahashi K, Fukuda H, Wakabayashi H. 2000. Waterborne transmission of Flavobacterium psychrophilum in coho salmon eggs. Fish Pathol. 35:25–28. doi:10.3147/jsfp.35.25

[12] Rangdale RE, Richards RH, Alderman DJ. 1996. Isolation of Cytophaga psychrophila, causal agent of rainbow trout fry syndrome (RTFS) from reproductive fluids and egg surfaces of rainbow trout (Oncorhynchus mykiss). Bull Eur Assoc Fish Pathol 16:2–63.

[13] Madetoja J, Dalsgaard I, Wiklund T. 2002. Occurrence of Flavobacterium psychrophilum in fish-farming environments. Dis Aquat Org. 52:109–118. doi:10.3354/dao052109 12542087

[14] Madetoja J, Nyman P, Wiklund T. 2000. Flavobacterium psychrophilum, invasion into and shedding by rainbow trout Oncorhynchus mykiss. Dis Aquat Organ 43:27–38. doi:10.3354/dao043027 11129378

[15] Knupp C, Wiens GD, Faisal M, Call DR, Cain KD, Nicolas P, Van Vliet D, Yamashita C, Ferguson JA, Meuninck D, Hsu H-M, Baker BB, Shen L, Loch TP. 2019. Large-scale analysis of Flavobacterium psychrophilum multilocus sequence typing genotypes recovered from North American Salmonids indicates that both newly identified and recurrent clonal complexes are associated with disease. Appl Environ Microbiol 85:e02305-18. doi:10.1128/AEM.02305-18 30658978 PMC6414368

[16] Knupp C., Kiupel M, Brenden TO, Loch TP. 2021. Host-specific preference of some Flavobacterium psychrophilum multilocus sequence typing genotypes determines their ability to cause bacterial coldwater disease in coho salmon (Oncorhynchus kisutch). J Fish Dis 44:521–531. doi:10.1111/jfd.13340 33476403

[17] Nicolas P, Mondot S, Achaz G, Bouchenot C, Bernardet J-F, Duchaud E. 2008. Population structure of the fish-pathogenic bacterium Flavobacterium psychrophilum. Appl Environ Microbiol 74:3702–3709. doi:10.1128/AEM.00244-08 18424537 PMC2446562

[18] Knupp C., Faisal M, Wiens GD, Brenden TO, Loch TP. 2021. In vivo experiments provide evidence that Flavobacterium psychrophilum strains belonging to multilocus sequence typing clonal complex ST191 are virulent to rainbow trout (Oncorhynchus mykiss). J Aquat Anim Health 33:190–195. doi:10.1002/aah.10140 34288128

[19] Sundell K, Landor L, Nicolas P, Jørgensen J, Castillo D, Middelboe M, Dalsgaard I, Donati VL, Madsen L, Wiklund T. 2019. Phenotypic and genetic predictors of pathogenicity and virulence in Flavobacterium psychrophilum Front Microbiol 10:1711. doi:10.3389/fmicb.2019.01711 31396199 PMC6668605

[20] Van Vliet D, Wiens GD, Loch TP, Nicolas P, Faisal M. 2016. Genetic diversity of Flavobacterium psychrophilum isolates from three Oncorhynchus spp. in the United States, as revealed by multilocus sequence typing. Appl Environ Microbiol 82:3246–3255. doi:10.1128/AEM.00411-16 27016570 PMC4959235

[21] Avendaño-Herrera R., Houel A, Irgang R, Bernardet J-F, Godoy M, Nicolas P, Duchaud E. 2014. Introduction, expansion, and coexistence of epidemic Flavobacterium psychrophilum lineages in Chilean fish farms. Vet Microbiol 170:298–306. doi:10.1016/j.vetmic.2014.02.009 24636160

[22] Fujiwara-Nagata E, Chantry-Darmon C, Bernardet J-F, Eguchi M, Duchaud E, Nicolas P. 2013. Population structure of the fish pathogen Flavobacterium psychrophilum at whole-country and model river levels in Japan. Vet Res 44:34. doi:10.1186/1297-9716-44-34 23682575 PMC3660162

[23] Li S, Chai J, Cao Y, Knupp C, Wang D, Nicolas P, Chen F, Liu H, Lu T, Loch TP. 2021. Characterization and molecular epidemiological analysis of Flavobacterium psychrophilum recovered from diseased salmonids in China. Microbiol Spectr 9:e00033021. 10.1128/Spectrum.00330-21 PMC8557942 34523994

[24] Nilsen Hanne, Sundell K, Duchaud E, Nicolas P, Dalsgaard I, Madsen L, Aspán A, Jansson E, Colquhoun DJ, Wiklund T. 2014. Multilocus sequence typing identifies epidemic clones of Flavobacterium psychrophilum in Nordic countries. Appl Environ Microbiol 80:2728–2736. doi:10.1128/AEM.04233-13 24561585 PMC3993282

[25] Siekoula-Nguedia C, Blanc G, Duchaud E, Calvez S. 2012. Genetic diversity of Flavobacterium psychrophilum isolated from rainbow trout in France: predominance of a clonal complex. Vet Microbiol 161:169–178. doi:10.1016/j.vetmic.2012.07.022 22871298

[26] Strepparava N, Nicolas P, Wahli T, Segner H, Petrini O. 2013. Molecular epidemiology of Flavobacterium psychrophilum from Swiss fish farms. Dis Aquat Org. 105:203–210. doi:10.3354/dao02609 23999704

[27] Madetoja J, Nystedt S, Wiklund T. 2003. Survival and virulence of Flavobacterium psychrophilum in water microcosms. FEMS Microbiol Ecol 43:217–223. doi:10.1111/j.1574-6941.2003.tb01061.x 19719682

[28] Madetoja J, Wiklund T. 2002. Detection of the fish pathogen Flavobacterium psychrophilum in water from fish farms. Syst Appl Microbiol 25:259–266. doi:10.1078/0723-2020-00105 12353881

[29] Strepparava N, Wahli T, Segner H, Petrini O. 2014. Detection and quantification of Flavobacterium psychrophilum in water and fish tissue samples by quantitative real time PCR. BMC Microbiol. 14:105. doi:10.1186/1471-2180-14-105 24767577 PMC4005812

[30] Marancik DP, Wiens GD. 2013. A real-time polymerase chain reaction assay for identification and quantification of Flavobacterium psychrophilum and application to disease resistance studies in selectively bred rainbow trout Oncorhynchus mykiss. FEMS Microbiol Lett 339:122–129. doi:10.1111/1574-6968.12061 23227879

[31] Standish I, Leis E, Schmitz N, Credico J, Erickson S, Bailey J, Kerby J, Phillips K, Lewis T. 2018. Optimizing, validating, and field testing a multiplex qPCR for the detection of amphibian pathogens. Dis Aquat Org. 129:1–13. doi:10.3354/dao03230 29916388

[32] Griffin MJ, Mauel MJ, Greenway TE, Khoo LH, Wise DJ. 2011. A real-time polymerase chain reaction assay for quantification of Edwardsiella ictaluri in catfish pond water and genetic homogeneity of diagnostic case isolates from Mississippi. J Aquat Anim Health 23:178–188. doi:10.1080/08997659.2011.637006 22372245

[33] Griffin MJ, Pote LM, Camus AC, Mauel MJ, Greenway TE, Wise DJ. 2009. Application of a real-time PCR assay for the detection of Henneguya ictaluri in commercial channel catfish ponds. Dis Aquat Org. 86:223–233. doi:10.3354/dao02079 20066957

[34] Knupp, C. 2023. PhD thesis. intraspecific Flavobacterium psychrophilum diversity as a factor in bacterial coldwater disease ecology and management. Michigan State University, East Lansing, MI.

[35] Kralik P, Slana I, Kralova A, Babak V, Whitlock RH, Pavlik I. 2011. Development of a predictive model for detection of Mycobacterium avium subsp. paratuberculosis in faeces by quantitative real time PCR. Vet Microbiol 149:133–138. doi:10.1016/j.vetmic.2010.10.009 21075565

[36] Lebuhn M, Effenberger M, Garcés G, Gronauer A, Wilderer PA. 2004. Evaluating real-time PCR for the quantification of distinct pathogens and indicator organisms in environmental samples. Water Science and Technology 50:263–270. doi:10.2166/wst.2004.0065 15318520

[37] Ricchi M, Savi R, Bolzoni L, Pongolini S, Grant IR, De Cicco C, Cerutti G, Cammi G, Garbarino CA, Arrigoni N. 2016. Estimation of Mycobacterium avium subsp. paratuberculosis load in raw bulk tank milk in Emilia-Romagna region (Italy) by qPCR. Microbiologyopen 5:551–559. doi:10.1002/mbo3.350 26991108 PMC4985589

[38] Slana I, Kralik P, Kralova A, Pavlik I. 2008. On-farm spread of Mycobacterium avium subsp. paratuberculosis in raw milk studied by IS900 and F57 competitive real time quantitative PCR and culture examination. Int J Food Microbiol 128:250–257. doi:10.1016/j.ijfoodmicro.2008.08.013 18824269

[39] Stoeckel DM, Stelzer EA, Dick LK. 2009. Evaluation of two spike-and-recovery controls for assessment of extraction efficiency in microbial source tracking studies. Water Res. 43:4820–4827. doi:10.1016/j.watres.2009.06.028 19589555

[40] van Tongeren SP, Degener JE, Harmsen HJM. 2011. Comparison of three rapid and easy bacterial DNA extraction methods for use with quantitative real-time PCR. Eur J Clin Microbiol Infect Dis 30:1053–1061. doi:10.1007/s10096-011-1191-4 21311936 PMC3181010

[41] Kralik P, Ricchi M. 2017. A basic guide to real time PCR in microbial diagnostics: definitions, parameters, and everything. Front Microbiol 8:108. doi:10.3389/fmicb.2017.00108 28210243 PMC5288344

[42] Sundell K, Wiklund T. 2015. Characteristics of epidemic and sporadic Flavobacterium psychrophilum sequence types. Aquaculture 441:51–56. doi:10.1016/j.aquaculture.2015.02.010

[43] Kunttu HMT, Valtonen ET, Jokinen EI, Suomalainen L-R. 2009. Saprophytism of a fish pathogen as a transmission strategy. Epidemics 1:96–100. doi:10.1016/j.epidem.2009.04.003 21352756

[44] Churchman EM, Parello G, Lange MD, Farmer BD, LaFrentz BR, Beck BH, Liles MR. 2022. Draft genome sequences of Flavobacterium covae strains LSU-066-04 and LV-359-01. Microbiol Resour Announc 11:e0035222. doi:10.1128/mra.00352-22 35703564 PMC9302162

[45] Rose AS, Ellis AE, Munro ALS. 1989. The infectivity by different routes of exposure and shedding rates of Aeromonas salmonicida subsp. Salmonicida in Atlantic salmon, Salmo salar L., held in sea water. J. Fish Dis 12:573–578. doi:10.1111/j.1365-2761.1989.tb00566.x

[46] Knupp C, Loch TP. 2023. Immersion challenge of three salmonid species (family Salmonidae) with three multilocus sequence typing variants of Flavobacterium psychrophilum provides evidence of differential host specificity. J Fish Dis. doi:10.1111/jfd.13889 PMC12285751 37974459

[47] Lorenzen E, Olesen NJ. 1997. Characterization of isolates of Flavobacterium psychrophilum associated with coldwater disease or rainbow trout fry syndrome ii: serological studies. Dis Aquat Org. 31:209–220. doi:10.3354/dao031209

[48] Rochat T, Fujiwara-Nagata E, Calvez S, Dalsgaard I, Madsen L, Calteau A, Lunazzi A, Nicolas P, Wiklund T, Bernardet J-F, Duchaud E. 2017. Genomic characterization of Flavobacterium psychrophilum serotypes and development of a multiplex PCR-based serotyping scheme. Front Microbiol 8:1752. doi:10.3389/fmicb.2017.01752 28955320 PMC5601056

[49] Delamater PL, Street EJ, Leslie TF, Yang YT, Jacobsen KH. 2019. Complexity of the basic reproduction number (R_0_). Emerging Infect. Dis 25:1–4. doi:10.3201/eid2501.171901 PMC6302597 30560777

[50] Kavlick MF. 2018. Development of a universal internal positive control. Biotechniques 65:275–280. doi:10.2144/btn-2018-0034 30394127

[51] Brenden TO, Ivan LN, Loch TP. 2023. Reducing Flavobacterium psychrophilum transmission risk via hatchery-rearing practices: an individual-based modeling evaluation. Aquaculture 563:738868. doi:10.1016/j.aquaculture.2022.738868

